# Window-of-Opportunity Study Utilizing Preoperative Cetuximab in Patients with Head and Neck Cancer

**DOI:** 10.1158/2767-9764.CRC-26-0258

**Published:** 2026-10-06

**Authors:** Justine Y. Bruce, Tiffany A. Glazer, Liliana L. Berube, Colin A. Longhurst, Menggang Yu, Rong Hu, Aaron M. Wieland, Paul M. Harari, Gregory K. Hartig, Mari Iida, Ranjit K. Mehta, Theodore S. Lawrence, Mukesh K. Nyati, Michael J. Poellmann, Jiyoon Bu, Kwangok P. Nickel, Seungpyo Hong, Deric L. Wheeler, Randall J. Kimple

**Affiliations:** 1 Department of Medicine, University of Wisconsin School of Medicine and Public Health, Madison, Wisconsin.; 2 University of Wisconsin Carbone Cancer Center, University of Wisconsin School of Medicine and Public Health, Madison, Wisconsin.; 3 Department of Surgery, University of Wisconsin School of Medicine and Public Health, Madison, Wisconsin.; 4 Department of Human Oncology, University of Wisconsin School of Medicine and Public Health, Madison, Wisconsin.; 5 Department of Biostatistics and Medical Informatics, University of Wisconsin School of Medicine and Public Health, Madison, Wisconsin.; 6 Department of Pathology and Laboratory Medicine, University of Wisconsin School of Medicine and Public Health, Madison, Wisconsin.; 7 Department of Radiation Oncology, University of Michigan, Ann Arbor, Michigan.; 8 Division of Pharmaceutical Sciences, School of Pharmacy, University of Wisconsin, Madison, Wisconsin.; 9 Lachman Institute for Pharmaceutical Development and Wisconsin Center for NanoBioSystems, School of Pharmacy, University of Wisconsin, Madison, Wisconsin.

## Abstract

**Purpose:**

AXL is a receptor tyrosine kinase implicated in epidermal growth factor receptor resistance. The primary objective of this study was to test the hypothesis, generated in preclinical studies, that low AXL correlates with clinical response to cetuximab in patients with head and neck cancer (HNC). The secondary objectives were to further describe the feasibility and safety of preoperative administration of cetuximab.

**Patients and Methods:**

We performed a single-site, single-arm, open-label, window-of-opportunity study utilizing preoperative cetuximab in 15 patients with HNC. Patients received two doses of cetuximab and underwent resection of their tumors. Biopsies were used to assess AXL expression and changes in Ki67 staining and to establish patient-derived xenografts (PDX). Single-cell RNA sequencing (scRNA-seq) of treated xenograft tissue was used to assess differences between sensitive and resistant tumors.

**Results:**

Fifteen subjects were registered for treatment in the study, and fourteen completed treatment with cetuximab prior to surgical resection. No correlation between AXL expression at baseline and change in tumor size in response to cetuximab was observed. Preoperative cetuximab did not delay surgical resection, and no unexpected treatment-related adverse events were reported. scRNA-seq identified that cetuximab sensitivity is marked by transcriptional remodeling, whereas resistance is associated with stable human epidermal growth factor receptor signaling and elevated Tyro, AXL, and MerTK (TAM) activity.

**Conclusions:**

Delivery of preoperative cetuximab is safe, and a short course can be utilized to identify patients with HNC responding to single-agent cetuximab treatment.

**Significance:**

Preoperative cetuximab is safe and feasible in HNC, identifying early responders without delaying surgery. Although AXL was not validated as a resistance biomarker—possibly due to COVID-19–related early closure—concordance among patient outcomes, circulating tumor cells, and PDXs strongly supports this translational pipeline for future studies.

## Introduction

In 2006, the FDA approved cetuximab, an mAb against the epidermal growth factor receptor (EGFR), for the treatment of head and neck squamous cell carcinoma (HNSCC). The addition of cetuximab to standard radiation improves absolute survival rates for patients with newly diagnosed HNSCC by approximately 10% (1, 2). Cetuximab is also valuable in the metastatic/recurrent setting, in which approximately 36% of patients respond to cetuximab-based combination therapy (vs. 20% to platinum-fluorouracil alone; ref. 3). Although cetuximab offers a significant advance for many patients with HNSCC, nearly two thirds of patients with cancer expressing EGFR do not respond to cetuximab-based therapy. The response rate to cetuximab alone for patients with metastatic HNSCC is approximately 10% to 20%, highlighting the need for improved treatments (4, 5). Virtually all patients who initially respond eventually become refractory to cetuximab. Intrinsic and acquired resistance to cetuximab is, therefore, a significant clinical problem in the treatment of HNSCC (6). Enhancing the effectiveness and survival impact of cetuximab would be highly valuable for the population of patients with HNSCC.

As the only molecular-targeting agent approved in head and neck cancer (HNC) therapy, a detailed understanding of how HNSCCs become refractory to cetuximab therapy is central to further refining the impact of this molecular agent and its clinical efficacy. Furthermore, the development of biomarkers leading to the identification of patients who will be responsive to cetuximab therapy is of high significance. Resistance to cetuximab has been explored, and several mechanisms of resistance have been reported, including altered angiogenesis (7–9), increased EGFR activity (10), increased expression of human epidermal growth factor receptor (HER) family ligands (11, 12), decreased EGFR degradation (10, 13, 14), epithelial-to-mesenchymal shift (15), phosphatase and tensin homolog (PTEN) degradation (16), a role for the nuclear EGFR signaling network (12), activation of HER3 (10, 17–22), and overexpression of the AXL receptor tyrosine kinase (23, 24). The promising impact of antiangiogenic agents combined with radiation or with cetuximab in HNSCC models and early clinical trial data suggests additional potential approaches to influence cetuximab response (25–28).

AXL is a member of the Tyro, AXL, and MerTK (TAM) family of receptors and has been shown to mediate resistance to several treatment modalities, including radiation (24, 29–32), chemotherapy (24, 31, 33–35), and molecular-targeted therapy (23, 31, 36–46). Furthermore, we identified that tyrosine 821 of AXL mediates resistance to cetuximab by the activation of c-ABL kinase in HNSCC and that the targeting of both EGFR and c-ABL leads to a robust antitumor response (31).

Based on these data, we hypothesized that low AXL expression could be predictive of response to cetuximab. To improve our understanding of AXL-mediated cetuximab resistance and to test this hypothesis, we performed a window-of-opportunity clinical study utilizing preoperative cetuximab in patients with HNC. Secondary objectives were to further describe the feasibility and safety of preoperative cetuximab. The collection of matched pre- and post-cetuximab tumor specimens could allow us to assess immediate molecular changes induced by cetuximab. The identification of adaptive resistance mechanisms before the confounding effects of surgery would be very beneficial. In this study, we used a previously published tissue microarray (TMA; ref. 47) containing pre- and post-cetuximab tissue samples, in addition to samples obtained as part of this study, to compare the expression of the putative resistance marker AXL and response as measured by both clinical response and change in the Ki67 proliferation index. Our novel circulating tumor cell (CTC) assay (48–51) was used to identify changes in CTCs induced by cetuximab. Furthermore, patient-derived xenografts (PDX) were established to test the concordance of response *in vivo* with patient response and investigate a novel combination therapy based on a proposed mechanism of resistance using single-cell RNA sequencing (scRNA-seq).

## Patients and Methods

### Sex as a biological variable

In the clinical trial, participants included both females and males. In the animal studies, females were used as subjects. It is unknown whether the findings are relevant for male mice. In both the clinical trial and the animal studies, sex was not considered a biological variable.

### Window-of-opportunity study: patients, study design, statistical analysis, and treatment

The clinical trial was a single-site, single-arm, open-label, window-of-opportunity study evaluating cetuximab in adult patients (age ≥ 18 years) with mucosal HNSCC who were anticipated to undergo surgical resection of their tumor. Subjects with locally advanced cutaneous HNSCC were excluded from this trial. Patients with newly diagnosed cancer, a presumed cancer diagnosis, or those who presented with recurrent disease were eligible for the screening and biopsy portion of the clinical trial if they were clinically deemed to be candidates for surgical resection. Subjects who were scheduled to undergo a standard-of-care diagnostic biopsy were eligible to consent and undergo the research biopsy if the research biopsy could be done concurrently with the standard-of-care biopsy. If a diagnosis other than mucosal HNSCC was made, these patients were discontinued from the study prior to any treatment with cetuximab. Patients who were diagnosed with mucosal HNSCC were eligible for the treatment portion of this study with cetuximab.

**Figure 1.**
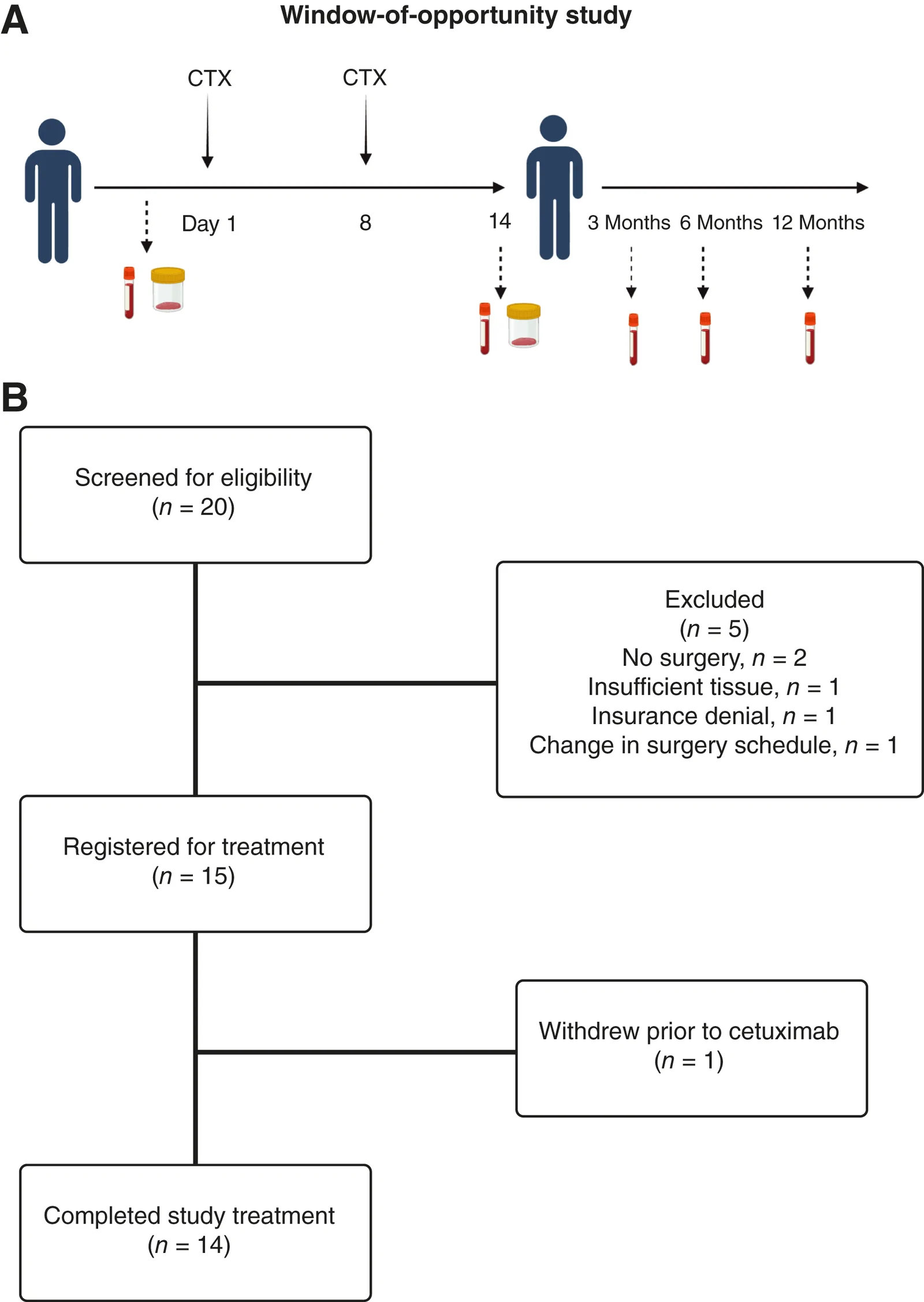
**A,** Window-of-opportunity scheme. **B,** CONSORT diagram of the window-of-opportunity study.

All patients underwent a baseline research biopsy (in the clinic or at the time of panendoscopy) and a staging workup with cross-sectional imaging of the neck, typically a CT scan with intravenous contrast. Patients received two doses of cetuximab within a 14-day period between the time of their baseline research biopsy and their scheduled surgery for resection of their tumor (Fig. 1A). Treatment with 2 weeks of preoperative cetuximab did not delay curative surgical resection. Tumor measurements were obtained by physical exam at baseline and on the day of surgery. Imaging was not used for tumor measurements in this study but was used for appropriate staging.

Patients received cetuximab dosing according to the package insert (dose #1: 400 mg/m^2^ intravenously over 2 hours; dose #2: 250 mg/m^2^ intravenously over 1 hour). No dose reductions of cetuximab were allowed although the rate of infusion of the drug could be reduced at the discretion of the investigator, as per the standard-of-care infusion rate modifications noted in the FDA prescribing information for cetuximab.

The surgical resection was performed as a standard-of-care therapy for this study population. Patients underwent definitive standard-of-care surgery within 4 weeks of the first protocol dose of cetuximab. The second research biopsy was collected from the remnant surgical specimen after completion of the required diagnostic pathologic review.

The study’s primary objective was to test the hypothesis that low AXL correlates with clinical response to cetuximab in patients with HNC. The primary objective was only assessed in patients treated under this study. The study’s secondary objective was to further describe the safety of preoperative administration of cetuximab. Correlative objectives included evaluating AXL expression with change in Ki67 following cetuximab in patients with HNC; examining other putative markers of cetuximab sensitivity such as HER3 and change in CTCs; and establishing a panel of PDXs from patients with known sensitivity or resistance to cetuximab to allow us to evaluate hypothesized mechanisms.

The original study design planned to enroll 36 patients, powered on the basis of the primary study endpoint: the clinical response rate according to RECIST version 1.1 (52) in low-AXL versus high-AXL patients. Based on the assumption that one in three patients responds to cetuximab and one in three has high AXL expression, this sample size provided 90% power to detect a difference between a response rate of 20% in the high AXL (2+ or 3+) group versus 65% in the low AXL (0 or 1+) group, with an expected ratio of 2:1 low- versus high-AXL expression. One-sided type I error was fixed at 5%.

The study was conducted in accordance with the Institutional Review Board (IRB)–approved protocol, the International Conference on Harmonisation, Good Clinical Practice Guidelines, and the Declaration of Helsinki. All patients were provided written informed consent; the study was approved by the local ethics committee/IRB (UW-IRB: 2018-1232, approved December 11, 2018) and was registered at clinicaltrials.gov (NCT03769311).

### CTC liquid biopsy

CTCs were collected and enumerated from peripheral blood samples according to a previously described process (48–51). CTCs were collected at baseline, after two doses of cetuximab, and immediately prior to surgery, and then 1, 3, and 12 months postoperatively. Briefly, mononuclear cells were purified from 6 to 10 mL of whole blood collected in sodium heparin tubes using Ficoll separation. Cells were resuspended to 0.2 mL and pumped through a flow chamber containing a surface functionalized with recombinant human E-selectin (R&D Systems, #7224-ES) and a mixture of polyclonal antibodies [targeting EpCAM (AF960, RRID: AB_355745), EGFR (AF231, RRID: AB_355220), and HER2 (AF1129, RRID: AB_354617), R&D Systems] conjugated to generation 7 poly(amidoamine) dendrimers (Dendritech) for the purification of CTCs. The surfaces were fixed, permeabilized, and stained for cytokeratin (Abcam, ab9377, RRID: AB_307222) and CD45 (BD Biosciences, 555480, RRID:AB_395872), followed by goat anti-rabbit Alexa Fluor 647 (Thermo Fisher Scientific, SA5-10378-AFP647, RRID:AB_3719777), goat anti-mouse Alexa Fluor 488 (Thermo Fisher Scientific, SA5-10370-AFP488, RRID:AB_3668616), and 4′,6-diamidino-2-phenylindole. Surfaces were scanned with a 10× objective using wide-field fluorescent microscopy (ZEISS Axiovert). A machine learning algorithm was used to enumerate cells containing nuclei that were positive for cytokeratin and negative for CD45.

### Animal studies

All animal experiments were conducted under an American Association for Laboratory Animal Care–accredited Institutional Animal Care and Use Committee–approved protocol. PDX models were established from pretreatment biopsies as we have previously described (53–55). Briefly, to establish PDXs, disaggregated tumor fragments were implanted into female NOD/SCID gamma mice (4–6 weeks old) obtained from the University of Wisconsin Research Animal Model Core (RRID:SCR_024797). Two PDXs were established from patients treated in this study (UW-SCC 185 and UW-SCC 194). PDXs were cryopreserved as previously described (54). *In vivo* testing was performed by implanting PDXs into the dorsal flank of female nude mice. Tumor volume was measured using a digital caliper, and when tumors reached a volume of 200 mm^3^, mice were randomized into treatment groups for animal study 1: (i) vehicle + immunoglobulin G (IgG); (ii) the Axl inhibitor, R428; (iii) cetuximab; or (iv) cetuximab + R428. Cetuximab or IgG was administered by i.p. injection (0.2 mg twice weekly). R428 or vehicle control was administered by oral gavage (25 mg/kg twice daily, 5 days per week). To examine the impact of treatment on response biomarkers, tumors were harvested at both an early time point (day 4 after the start of treatment) and a late time point (day 27 after the start of treatment). Tumors were collected within 3 hours of the last treatment for analysis of biomarkers of response.

An additional study, animal study 2, using PDXs established from UW-SCC 185 and UW-SCC 194, was conducted. Implantation and randomization were completed as described above but for only two treatment groups: (i) vehicle or (ii) cetuximab. Cetuximab was administered by i.p. injection (0.2 mg twice weekly). Tumors were harvested 14 days after the start of treatment and within 4 hours of the last treatment for analysis of biomarkers of response.

### IHC

Tumors were processed for IHC analysis as described previously (31). Ki67 (Cell Signaling Technology, #9027, RRID:AB_2636984, 1:400) and AXL (Abcam, Ab219651) antibodies were used and detected using the VECTASTAIN Universal Kit/HRP (Vector Lab) according to the manufacturer’s recommendations. Paraffin-embedded tissues mounted on slides were heated in a 10 mmol/L Tris-EDTA buffer (pH 9.0) for AXL and a 10 mmol/L citrate buffer (pH 6.0) for Ki67 in a Decloaking Chamber. The anti-AXL antibody (1:2,000) and Ki67 antibody (1:400) were used for staining. All antibodies were then visualized with 3,3′-diaminobenzidine chromagen, and hematoxylin was used for counterstaining. Patient samples were stained for Ki67 and reviewed by a board-certified surgical pathologist (R. Hu) by counting the percentage of tumor cells with positive nuclear staining compared with the total number of tumor cells. At least 500 tumor cells were analyzed. Cytoplasmic and membranous AXL expression was evaluated throughout the entire available tumor on the slides. In most cases, staining intensity correlated with the percentage of positive tumor cells; therefore, a combined semiquantitative scoring system was used: 0, no staining or staining in <10% of tumor cells; 1+, weak staining in <25% of tumor cells; 2+, moderate staining in 25% to 75% of tumor cells; 3+, moderate staining in >75% of tumor cells; and 4+, strong staining in >75% of tumor cells. Cases scored as 2+ or higher were considered to show high AXL expression. For PDX tumor samples (4 samples/group), images were taken at a magnification of 20× and were quantified in three separate areas/section by counting the number of positive cells using ImageJ.

Due to reduced accrual, in part related to the COVID-19 pandemic, we obtained a TMA from a previously published study reporting a pair of window-of-opportunity studies utilizing a similar approach of cetuximab treatment at the University of Michigan (NCT01663259, NCT00904345; ref. 47) to assess correlative objectives. In this study, patients received one dose of cetuximab prior to definitive chemoradiation. Pretreatment and post-cetuximab biopsies were available from a subset of patients. These samples had been previously stained for Ki67 but, to ensure consistency, were independently evaluated by our pathologist (R. Hu). Expression of AXL was assessed as described above.

### Generation of single-cell gene expression libraries

scRNA-seq was performed on tumors harvested from animal study 2. Biological replicates (*n* = 10 for UW-SCC 185 and *n* = 12 for UW-SCC 194) were combined prior to dissociation to encompass biological heterogeneity. Dissociation from solid tumor to single-cell suspension was performed as described previously (56). High-quality cell suspensions were prepared with an average viability of more than 75%. scRNA-seq libraries were constructed using the PIPseq 3′ Single Cell RNA Kit v4.0 Plus (T20 for UW-SCC 185 and T2 for UW-SCC 194), following the manufacturer’s protocol. Libraries were sequenced on an Illumina NovaSeq X sequencer with a sequencing depth of at least 20,000 reads per cell.

### Data processing of scRNA-seq libraries

Gene-barcode matrices were generated using the PIPseeker program, which utilized STAR to map reads to the human and mouse genome (GRCh38.p13 + GRCm39) downloaded directly from Fluent BioSciences.

In RStudio (R version 4.40), raw gene-barcode matrices were imported for two PDXs: UW-SCC 185 (cetuximab-sensitive) and UW-SCC 194 (cetuximab-resistant), each with vehicle control and cetuximab-treated samples. For UW-SCC 185, two sequencing batches per treatment were included; UW-SCC 194 included one batch per treatment. Mixed-species reads were assessed by calculating the proportion of human and mouse transcripts per cell. Cells with at least 90% human reads were retained, and mouse genes were removed. Gene names were standardized by removing species prefixes. Remaining cells were filtered based on quality control thresholds: the number of genes detected between 200 and 5,000, the number of UMI counts between 500 and 20,000, and the mitochondrial percentage <10%. Genes expressed in fewer than five cells were excluded.

Downstream analysis was performed using the Seurat package (version 5.3.0). For each treatment group, libraries were merged and randomly downsampled to 3,000 cells to ensure a balanced representation. Expression values were normalized by scaling each cell’s total transcript count to a common value and applying a log transformation to stabilize variance across genes. Highly variable genes were identified by calculating the mean and variance for each gene across cells and selecting the top 2,000 genes with the highest standardized variance. These genes were used for downstream dimensionality reduction and clustering. Expression values for these genes were then centered and scaled so that each gene had a mean of zero and unit variance, ensuring comparability across features. Principal component analysis was performed on the scaled data to reduce dimensionality. The number of components retained for downstream analysis was chosen based on the elbow plot and the proportion of total variation captured by each component, ensuring that most of the variability in the dataset was represented while avoiding the inclusion of components that contributed little additional information. Clusters were identified by computing a shared nearest neighbor graph and applying a community detection algorithm with a resolution of 0.6. For both vehicle- and cetuximab-treated UW-SCC 185 samples, integration was performed using Harmony (version 1.2.3) with a θ of 50 to remove batch effects due to multiple sequencing runs. Cell types were annotated for each PDX treatment group using SingleR (version 2.8.0) and the Human Primary Cell Atlas as its reference. Epithelial cells were subsetted; treatment groups were combined for each patient’s PDX, and the previous normalization through integration steps was repeated. With epithelial-only clusters, Uniform Manifold Approximation and Projection for Dimension Reduction for each PDX was generated with a resolution of 0.6 using previously identified principal components and grouped by treatment.

PDX datasets were merged, log-normalized, and scaled before joining layers. Average expression of TAM receptor genes was calculated across sample groups using AverageExpression in Seurat. Genewise values were scaled (*z*-score per gene) prior to heatmap visualization with ComplexHeatmap (version 2.22.0). Expression of HER family receptors and ligand genes was visualized using Seurat’s DotPlot function, grouping cells by PDX treatment category. Dot size represents the fraction of expressing cells, whereas dot color reflects average scaled expression (blue–white–red gradient). Differential expression analysis of HER family genes and ligands was performed using Seurat’s FindMarkers() function with parameters logfc.threshold = 0.25 and min.pct = 0.1. Three comparisons were evaluated: cetuximab-treated versus vehicle control in the sensitive PDX (UW-SCC 185), cetuximab-treated versus vehicle control in the resistant PDX (UW-SCC 194), and baseline comparison between resistant and sensitive vehicle controls. Additionally, genome-wide differential expression was assessed without restriction to predefined features, using the same parameters. Average expression of genes in MAPK, PI3K/AKT, and STAT3 pathways was calculated across sample groups using AverageExpression in Seurat (57). Gene-wise values were scaled (*z*-score per gene) prior to heatmap visualization with ComplexHeatmap (version 2.22.0; ref. 58). Gene set enrichment analysis (GSEA) was performed with a subset of CP:BIOCARTA, filtering for relevant pathways including genes related to TAM and HER families and for sets with more than three genes. Rankings were calculated as sign(log_2_FC) × −log_10_(*P* value), where FC indicates fold change, and adjusted using the Benjamini–Hochberg correction [*P*.adjust(method = “BH”)]. Top pathways were visualized as dot plots with ggplot(). Overlapping pathways with the hallmark gene set were identified using the GSEA Compute Overlaps tool.

### Statistics

Statistical analysis of the clinical trial is provided in the above section. To estimate the differences in tumor growth curves between the treatment groups for both PDXs, a linear mixed model was fit to the volume data on the natural log scale using the “lme4” package in R. The fixed effect portion of each model included an interaction between time and treatment group, and each model included both a random intercept and slope at the animal level. Approximate model *P* values were calculated using the Kenward–Roger approach. A graphical analysis of the residuals revealed no apparent departure from model assumptions, and the random effects were found to be normal in distribution (59, 60).

## Results

### Clinical feasibility and safety of preoperative cetuximab

#### Patient demographics and accrual

Between February 26, 2019, and December 15, 2021, a total of 20 subjects consented to participate in the study (Fig. 1B). Five subjects were not eligible to proceed with study treatment (two subjects not pursuing surgical resection, one subject with insufficient tissue, one subject with a lack of insurance approval, and one subject with a change in the surgical procedure date that precluded treatment on study). A total of 15 subjects were registered for treatment in the study. After registration in the study, one subject withdrew early prior to treatment with cetuximab. Fourteen subjects completed treatment with cetuximab (two doses according to the trial protocol) prior to surgical resection (Table 1; Fig. 1A). All subjects who received preoperative therapy received both doses of cetuximab. The median time from the first dose of cetuximab to surgical resection was 14.5 days (range, 10–68 days). Accrual was a challenge during the conduct of the trial, due in part to the COVID-19 pandemic, which resulted in an institutional requirement to halt accrual. Due to reduced accrual, we utilized specimens from a separately reported study (University of Michigan) to examine Ki67 alterations and the expression of AXL. Demographic data on these patients have been previously reported (47).

**Table 1.** Patient demographics.

Characteristic	*N* = 14
Sex, *n* (%)	​
Female	4 (29)
Male	10 (71)
Age, median (range)	​
Age at baseline (years)	63 (24–88)
Race	​
White	14
Other	0
Ethnicity	​
Hispanic	0
Non-Hispanic	14
ECOG^a^ performance status, *n*	​
ECOG 0	12
ECOG 1	2
Primary tumor site	​
Oropharynx	0
Hypopharynx	0
Larynx	0
Oral cavity	14
Other	0
AJCC stage	​
I	1
II	4
III	3
IVA	5
IVB	1
IVC	0
Time from first dose of cetuximab to surgical resection, median (range)	​
Days	14.5 (10–68)
Doses of cetuximab received	​
Number of doses (SD)	2 (0)

Abbreviation: AJCC, American Joint Committee on Cancer.

a ECOG, Eastern Cooperative Oncology Group.

### Clinical response

#### Primary objective

**Figure 2.**
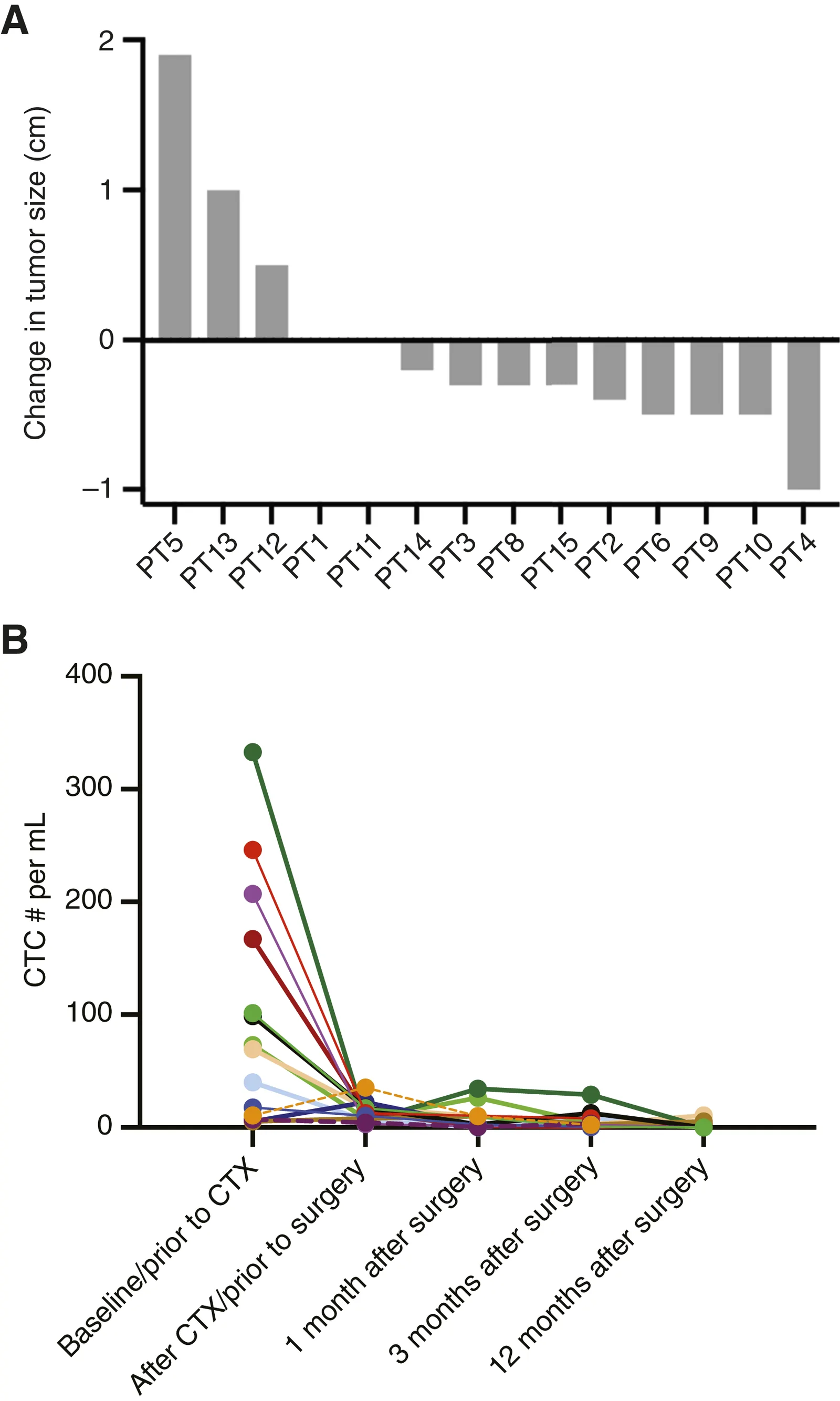
**A,** Waterfall plot for change in tumor size. **B,** CTCs detected before and following cetuximab (CTX) treatment and surgery. CTCs were collected at baseline, immediately prior to surgery after the second cetuximab administration, and then 1, 3, and 12 months after surgery. CTCs are expressed as the number detected per mL, and different lines/colors represent different patients (*n* = 11).

Analysis of the primary objective was limited by the accrual. Nine patients experienced a shrinkage of their tumor following the administration of cetuximab (defined as any decrease in tumor size), whereas three had tumor growth and two had no change in tumor size (Table 2; Fig. 2A). There was a correlation between the change in AXL expression relative to baseline (e.g., pre- and post-cetuximab) and the change in Ki67 in response to cetuximab, with a mean difference of −0.18 [95% confidence interval (CI), −0.32 to −0.03; *P* = 0.02; Supplementary Fig. S1].

**Table 2.** Baseline and post-cetuximab (CTX) IHC staining intensity for Ki67 (percentage of tumor cells positive), AXL (0 to 4+), and tumor size (cm).

UW18098 subject number	Baseline Ki67 index (%)	Post-CTX Ki67 (%)	Delta Ki67	Baseline AXL (0 to 4+)	Post-CTX AXL (0 to 4+)	Baseline tumor size (cm)	Post-CTX tumor size (cm)	Change from baseline to post-CTX
1	n/a^a^	56%	n/a	n/a	3+	1	1	0
2	26%	41%	15%	2+	3+	1.4	1	−0.4
3	26%	23%	−3%	2+	2+	1.2	0.9	−0.3
4	74%	63%	−11%	4+	4+	2.5	1.5	−1
5	24%	49%	25%	3+	4+	0.3	2.2	1.9
6	45%	26%	−19%	3+	3+	2.5	2	−0.5
7	Withdrew	​	​	​	​	​	​	​
8	39%	48%	9%	2+	2+	1.5	1.2	−0.3
9	77%	73%	−4%	2+	2+	3	2.5	−0.5
10	53%	73%	20%	3+	4+	3	2.5	−0.5
11	24%	46%	22%	1+	1+	1.5	1.5	0
12	56%	44%	−12%	2+	2+	2.5	3	0.5
13	19%	22%	3%	2+	3+	2	3	1
14	62%	60%	−2%	2+	2+	4.7	4.5	−0.2
15	66%	63%	−3%	2+	2+	2	1.7	−0.3
University of Michigan Cohort	​	​	​	​	​	​	​	​
078 UM01 T PRE	75%	34%	−41%	0	​	​	​	​
078 UM02 T PRE	15%	37%	22%	n/a	​	​	​	​
078 UM103 T PRE	18%	60%	42%	1+	​	​	​	​
078 UM106 T PRE	38%	50%	12%	2+	​	​	​	​
078 UM109 T PRE	48%	47%	−1%	2+	​	​	​	​
078 UM121 T PRE	21%	40%	19%	1+	​	​	​	​
078 UM122 T PRE	33%	50%	17%	1+	​	​	​	​
078 UM124 T PRE	23%	24%	1%	1+	​	​	​	​
078 UM126 T PRE	45%	27%	−18%	0	​	​	​	​
078 UM127 T PRE	21%	56%	35%	1+	​	​	​	​
078 UM128 T PRE	45%	57%	12%	n/a	​	​	​	​
078 UM131 T PRE	57%	56%	−1%	1+	​	​	​	​

a No tumor on the slide.

#### Adverse events during treatment

At the final analysis, no subjects experienced any grade 3 or worse all-cause adverse events. Adverse events occurred in 11 subjects out of the 14 subjects treated during the conduct of this study (Table 3). The most common treatment-related adverse events were acneiform rash [10 of 14 subjects (71%)] and headache [3 of 14 subjects (21%)]. Other adverse events reported included hypomagnesemia, nausea, and fatigue. No serious adverse events or unanticipated problems occurred during the conduct of the study. There were zero hospital readmissions for wound care or surgical complications attributed to cetuximab for all subjects enrolled in the study.

**Table 3.** Adverse events seen during treatment.

Event	Number of patients (%)	
	Grade 1^a^	Grade 2^a^
Any event	11 (79)	4 (29)
Acneiform rash	10 (71)	4 (29)
Headache	3 (21)	0 (0)
Nausea	2 (14)	0 (0)
Fatigue	2 (14)	0 (0)
Hypomagnesemia	1 (7)	0 (0)

a Adverse events assessed by NCI Common Terminology Criteria for Adverse Events, version 4.0, with possible grades ranging in severity from 1 (mild) to 5 (adverse event–related death). Grade 1 = mild, asymptomatic or mild symptoms, clinical or diagnostic observations only, and intervention not indicated; grade 2 = moderate, minimal, local, or noninvasive intervention indicated.

#### Correlative objectives

The Ki67 index (percentage of tumor cells that stain positive for Ki67) was decreased in seven patients, increased in six patients, and not evaluable in one patient due to insufficient tumor in the specimen at the pretreatment biopsy. Due to limitations of the small patient cohort, we obtained a TMA that had been previously described to expand our analysis cohort (47). Using these specimens, an additional 12 patients had sufficient pre- and post-cetuximab tissue to evaluate both Ki67 and AXL by IHC. Within this cohort, a decrease in the Ki67 index was seen in four patients, and an increase was seen in eight patients. No correlation was found between δ Ki67 and AXL expression in the pooled cohort (Supplementary Table S2; correlation of −0.107, *P* = 0.52). Each cohort analyzed individually also failed to demonstrate a correlation (Supplementary Table S2). Within the cohort for whom pre- and posttreatment biopsies were both able to be analyzed for AXL expression, an increase in AXL expression after cetuximab was correlated with a larger change in the Ki67 index compared with those in whom no change in AXL expression was seen (Supplementary Fig. S2, *P* = 0.03).

All patients had blood collected and assessed using a novel HNC-optimized CTC collection device. CTCs were detected in all patients. One patient had two draws with poor quality data and was thus excluded from analysis. A decrease in CTCs was seen in 11 patients after cetuximab administration (Fig. 2B). The average difference in the number of CTCs detected between baseline and after cetuximab was −85.764, with a 95% CI of −147.119 to −24.41 and a *P* value of 0.0099. Two patients developed recurrent/progressive disease in the first 12 months. Both had progression by the time of the 3-month postoperative visit. One demonstrated an increase in CTCs after cetuximab and detectable cells at the 1-month postoperative visit. The other had a low initial detectable CTC count that decreased by <50% after cetuximab and was trending up at the 3-month postoperative visit.

#### PDX establishment and molecular characterization

**Figure 3.**
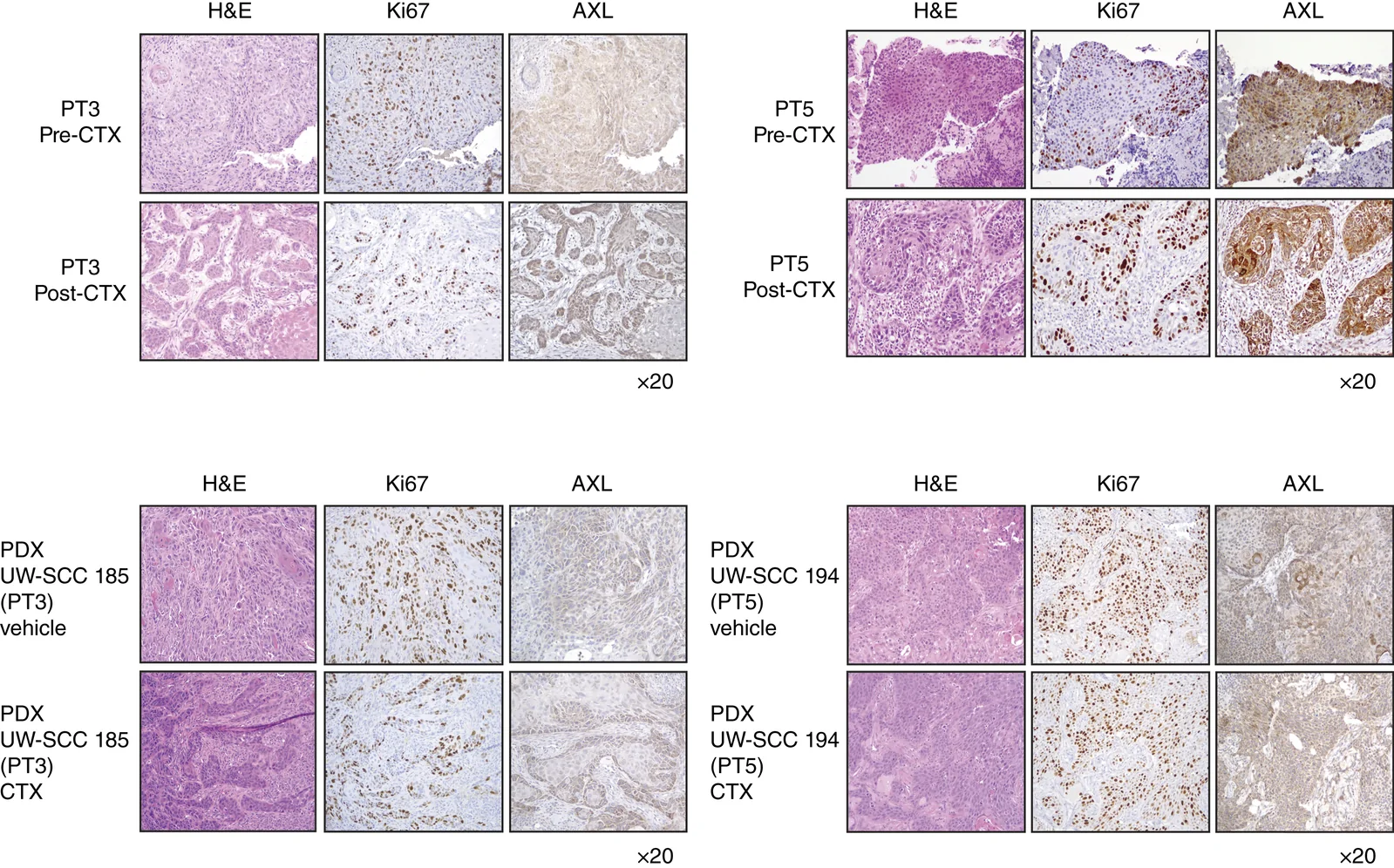
Histologic comparison of patient and PDX at baseline (pre-CTX) and after cetuximab (post-CTX) treatment. Hematoxylin and eosin (H&E), IHC for Ki67, and AXL for patient 3 (PT3) and patient 5 (PT5) before and after cetuximab are shown in the top two rows. The bottom two rows show similar staining of both PDX UW-SCC 185 from PT3 and PDX UW-SCC 194 from PT5 under vehicle conditions (top 3 boxes) and cetuximab conditions (bottom three boxes).

Two novel PDXs were established from pre-cetuximab biopsies. Although our goal was to establish PDXs from each patient, the establishment of PDXs was a lower priority for fresh tumor use, leading to a lower than anticipated success rate. Comparison of histology between the original patient sample and the PDX demonstrated similarity in tumor architecture and keratinization (Fig. 3). Similar staining of Ki67 both before and after cetuximab was seen in each case.

**Figure 4.**
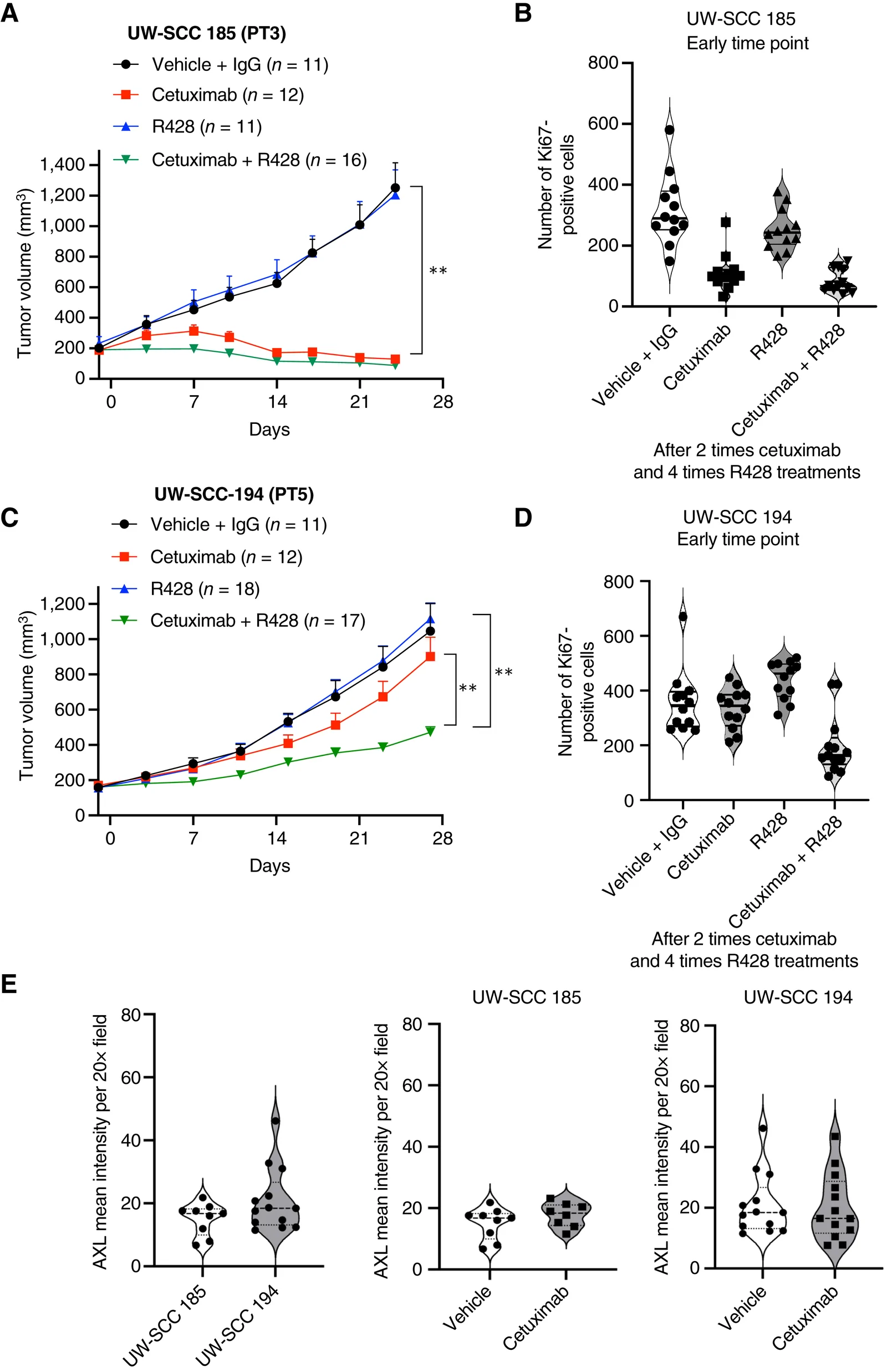
Response of PDX to vehicle + IgG, cetuximab, R428, and cetuximab + R428. **A,** Line graph shows tumor volume (mean ± SD) for UW-SCC 185 (PT3) PDX as a function of time since administration of vehicle + IgG (black circle, *n* = 11 tumors), cetuximab (red square, *n* = 12 tumors), R428 (blue triangle, *n* = 11 tumors), and cetuximab + R428 (green inverted triangle, *n* = 16 tumors). Volumes differed significantly between the vehicle + IgG and cetuximab conditions. **B,** Bar graph showing for the UW-SCC 185 PDX the mean (SD) number of Ki67-positive cells in each of the four conditions: vehicle + IgG, cetuximab, R428, and cetuximab + R428, after two doses of cetuximab and four doses of R428 treatments. **C,** Line graph shows tumor volume (mean ± SD) for UW-SCC 194 (PT5) PDX as a function of time since administration of vehicle + IgG (black circle, *n* = 11 tumors), cetuximab (red square, *n* = 12 tumors), R428 (blue triangle, *n* = 18 tumors), and cetuximab + R428 (green inverted triangle, *n* = 17 tumors). Volumes differed significantly between the cetuximab and cetuximab + R428 conditions and between the R428 and cetuximab + R428 conditions. **, *P* < 0.05. **D,** Bar graph showing for the UW-SCC 194 PDX the mean (SD) number of Ki67-positive cells in each of four conditions after two doses of cetuximab and four doses of R428: vehicle + IgG, cetuximab, R428, and cetuximab + R428. **E,** The graphs of AXL mean intensity in vehicle + IgG groups between UW-SCC 185 and UW-SCC 194 (left), vehicle + IgG vs. cetuximab groups in UW-SCC 185 (middle), and UW-SCC 194 (right).

PDXs were established in nude mice, and mice were treated with cetuximab with or without R428, a small molecule inhibitor of the AXL receptor tyrosine kinase (Fig. 4). Consistent with the response in the patient (PT3), UW-SCC 185 demonstrated a response to cetuximab (*P* < 0.001). In this PDX, the addition of R428 resulted in a small additional benefit (Fig. 4A). Analysis of Ki67 changes in each group mirrored the tumor growth data (Fig. 4B). A second PDX established from a patient (PT5) who did not respond to cetuximab, UW-SCC 194, also demonstrated little response to cetuximab (Fig. 4C). In this case, however, the addition of R428 resulted in a significant delay in tumor growth (Fig. 4C). Ki67 expression showed that tumors treated with cetuximab and R428 demonstrated reduced Ki67 positive staining compared with cetuximab or R428 treatment alone (Fig. 4D). The baseline of AXL expression in UW-SCC 194 (mean intensity: 20.97) was higher than that in UW-SCC 185 (mean intensity: 15.04; Fig. 4E). AXL expression showed a slight increase following cetuximab treatment in UW-SCC 194 and a slight decrease in UW-SCC 185; however, these changes were not statistically significant.

#### scRNA-seq analysis of sensitive versus resistant tumors

**Figure 5.**
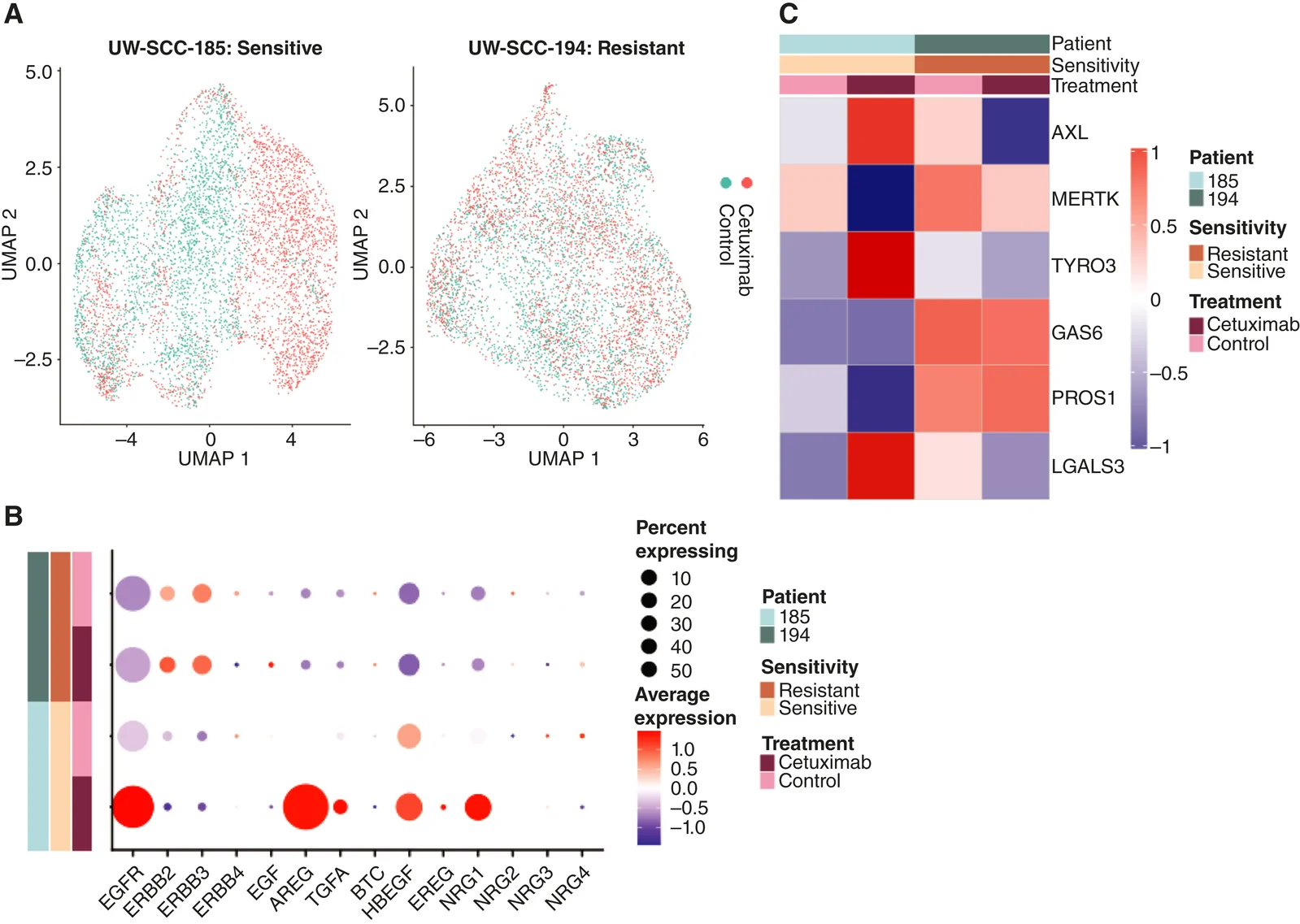
Transcriptional response of PDXs to vehicle and cetuximab. **A,** Uniform Manifold Approximation and Projection for Dimension Reduction (UMAP) of epithelial cells shows strong overlap of vehicle- and cetuximab-treated groups in UW-SCC 194 PDX in contrast to the clear separation of vehicle- and cetuximab-treated groups in UW-SCC 185 PDX, which corresponds to the difference in cetuximab response. **B,** Heatmap of TAM family receptors and ligands gene expression across sample groups, scaled by *z*-score per gene. **C,** Dot plot of HER family receptors and ligands showing average scaled expression [average expression shown = (average expression in group – mean expression across all groups)/(SD across all groups)] and percentage of expressing cells in each PDX under two conditions: vehicle and cetuximab.

To better understand resistance to cetuximab, we performed scRNA-seq on PDXs following treatment with cetuximab or vehicle control. Comparison of vehicle- and cetuximab-treated samples revealed strong overlap in UW-SCC 194 PDX (cetuximab-resistant) and clear separation in UW-SCC 185 PDX (cetuximab-sensitive), consistent with the patients’ known clinical responses (Fig. 5A).

To investigate the impact of cetuximab resistance on HER family signaling, we performed differential expression analysis of HER receptors and their ligands in UW-SCC 185 (cetuximab-sensitive) and UW-SCC 194 (cetuximab-resistant) PDX tumors (Fig. 5B). Baseline comparison of vehicle control samples revealed intrinsic signaling differences: UW-SCC 194 exhibited significantly higher *ERBB3* (log_2_FC ≈ +0.89, adj. *P* < 2.2 × 10^−28^) and *ERBB2* (+0.42, adj. *P* < 4.6 × 10^−8^) compared with UW-SCC 185, whereas UW-SCC 185 showed higher *AREG* (−2.07, adj. *P* < 3 × 10^−37^) and *NRG1* (−1.22, adj. *P* = 0.17), suggesting distinct baseline ligand–receptor profiles that may influence cetuximab response.

Following cetuximab treatment, the cetuximab-sensitive UW-SCC 185 PDX exhibited robust transcriptional reprogramming, with multiple *HER* ligands strongly upregulated (Fig. 5B): *AREG* (log_2_FC ≈ +2.34, adj. *P* < 1.2 × 10^−184^), *NRG1* (+1.79, adj. *P* < 3.2 × 10^−28^), *TGFA* (+1.31, adj. *P* < 6.6 × 10^−19^), and *EREG* (+1.87, adj. *P* = 0.0059). *EGFR* also increased significantly (+0.98, adj. *P* < 1.7 × 10^−32^). In contrast, *ERBB2* and *ERBB3* were modestly downregulated (−0.47 and −0.20, respectively), indicating a shift toward ligand-driven EGFR signaling. Conversely, UW-SCC 194 PDX showed minimal transcriptional changes upon cetuximab exposure. Among *HER*-related genes, only *EGF* displayed a modest increase (+0.63, *P* = 0.016), whereas *ERBB4* decreased slightly (−0.87, *P* = 0.023). All adjusted *P* values were nonsignificant, underscoring the stability of HER signaling in resistant tumors. Collectively, these findings highlight two distinct adaptive strategies: ligand induction and *EGFR* upregulation in sensitive tumors versus maintenance of *HER2/HER3* signaling in resistant tumors. These intrinsic and treatment-induced differences suggest that cetuximab resistance may involve reliance on alternative *HER* family members rather than dynamic ligand-driven feedback.

We next examined TAM family receptors and ligands, which revealed strikingly opposite changes in *AXL* expression (Fig. 5C). In the sensitive PDX, *AXL* shifted from slight downregulation in the vehicle control sample to strong upregulation after cetuximab treatment. Conversely, the resistant PDX shifted from slight upregulation in the vehicle control sample to strong downregulation after treatment. Increased baseline *AXL* expression in the resistant sample is consistent with other studies (23, 24), but the reduced expression post-cetuximab treatment may point to dynamic regulation of TAM signaling. Overall, most TAM genes and ligands were upregulated in the resistant PDX and downregulated in the sensitive PDX, supporting the role of TAM signaling in activating PI3K, MAPK, and STAT3 pathways and promoting resistance to EGFR-targeted therapy.

**Figure 6.**
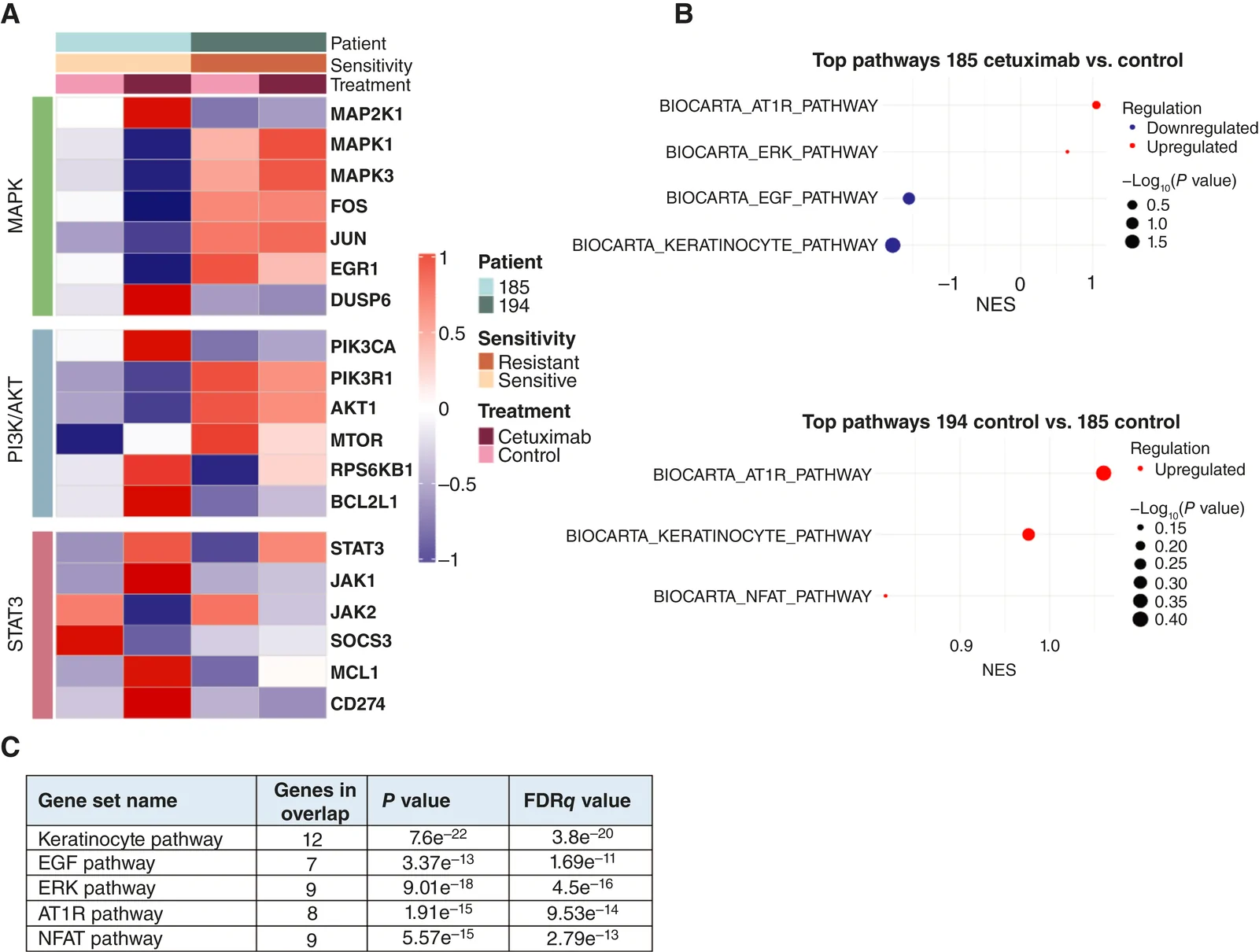
Transcriptional pathway alterations in PDXs following vehicle and cetuximab treatment. **A,** Heatmap of *z*-score scaled expression values for genes in MAPK, PI3K/AKT, and STAT3 pathways across sample groups. **B,** Dot plot of enriched signaling pathways identified by GSEA using a subset from CP:BIOCARTA. Dot size represents gene count, and color indicates adjusted *P* value. No significantly enriched signaling pathways were found for the comparison of UW-SCC 194 (PT5) PDX, so no plot is shown. **C,** Overlap analysis between enriched pathways and the PI3K/AKT/mTOR signaling pathway identified by the GSEA overlap tool.

Given these associations, we examined the expression of key genes within the MAPK, PI3K/AKT, and STAT3 pathways to determine whether downstream signaling reflected cetuximab response (Fig. 6A). Genes such as *MAP2K1*, *DUSP6* (MAPK pathway), *PIK3CA*, *MTOR*, *BCL2L1* (PI3K/AKT pathway), and *JAK1*, *SOCS3*, *CD274* (STAT3 pathway) exhibited minimal differences between the vehicle control samples of the two PDXs. However, following cetuximab treatment, the sensitive PDX displayed notable upregulation across multiple genes in all three pathways, whereas the resistant PDX showed little to no change. This pattern suggests that cetuximab sensitivity is associated with the activation of compensatory signaling through the MAPK, PI3K/AKT, and STAT3 pathways, whereas resistance is characterized by transcriptional stability in these downstream networks.

Pathway enrichment analysis revealed upregulation of the ERK and AT1R pathways and downregulation of the keratinocyte and EGF pathways in the sensitive PDX following cetuximab treatment (Fig. 6B). In contrast, no significant enrichment was observed between vehicle control and treated samples in the resistant PDX. Comparison of vehicle control samples showed higher baseline activity of the AT1R, keratinocyte, and NFAT pathways in the resistant PDX [Fig. 6B (bottom)]. Most enriched pathways were directly or indirectly linked to MAPK, PI3K/AKT, and STAT3 signaling. Computing each pathway’s overlap with the hallmark gene set identified that the PI3K/AKT/mTOR pathway had the greatest overlap with all enriched pathways previously identified, with 7 to 12 overlapping genes across enriched pathways (Fig. 6C). All overlapping enriched gene sets were statistically significant (hypergeometric test, FDR-adjusted *P* values).

## Discussion

This study was initiated to test a hypothesis generated based on preclinical studies demonstrating a key role for *AXL* in driving the response to cetuximab. Multiple preclinical models had demonstrated that tumors with high expression of *AXL* were resistant to cetuximab (23, 24). Slow accrual in this study, compounded by the COVID-19 pandemic, led to early closure and caused us to seek out additional sources of pre-cetuximab and matched post-cetuximab samples. Even after combining the two datasets, no clear correlation between *AXL* expression and response to cetuximab was seen although evidence of upregulation in a resistant PDX compared with a sensitive PDX was found using scRNA-seq. Furthermore, patients with higher *AXL* expression following cetuximab treatment exhibited a higher δ Ki67 value. Interestingly, we observed a substantial reduction in CTC counts despite limited changes in tumor size over the 14-day treatment period. This discrepancy may reflect differences in the biological processes captured by these endpoints. In addition, limited tissue availability and potential sampling error may have contributed to discrepancies among the molecular and clinical findings. Sampling bias and intratumoral heterogeneity are well-recognized challenges in translational studies of HNSCC. Future window-of-opportunity trials incorporating standardized pre- and posttreatment radiographic assessment may help better correlate molecular and CTC changes with treatment responses.

We confirmed previously reported safety data of preoperative cetuximab (61) and identified no unexpected adverse events during this short course of treatment. A similar approach is being taken in an ongoing window-of-opportunity study at our institution testing the combination of cetuximab plus imatinib in patients with HNSCC (NCT05816785).

Multiple correlative objectives were included in the study design. Insufficient tissue was available to perform immunostaining for HER3, highlighting a critical limitation of window-of-opportunity studies: small sample sizes. Although multiplex approaches could help overcome these limitations, they were not used in this work. The CTC assay demonstrated a decrease in CTCs following cetuximab in most patients, suggesting a potential discrepancy between the response seen clinically and that seen using circulating biomarkers. Most patients demonstrated a decrease in the number of detectable CTCs after only 2 weeks of cetuximab and a further decrease after surgical resection of their tumor. The PDX models developed during this study were faithful avatars of the response to cetuximab in each patient. Both PDXs demonstrated a benefit of adding R428 to cetuximab, consistent with our previously published data (24, 31).

Single-cell analysis revealed distinct transcriptional responses to cetuximab between a sensitive PDX (UW-SCC 185) and a resistant PDX (UW-SCC 194). Epithelial cell clustering showed a strong overlap between the vehicle- and cetuximab-treated samples in the resistant PDX but clear separation in the sensitive PDX, consistent with the clinical response.

HER family analysis indicated broader expression shifts in the sensitive PDX (8/13 genes) compared with those in the resistant PDX (3/14 genes), including marked *EGFR* changes. TAM receptor analysis revealed opposite adaptive changes in *AXL*: The sensitive PDX shifted from slight downregulation to strong upregulation, whereas the resistant PDX shifted from slight upregulation to strong downregulation. This dynamic reversal suggests differential pathway rewiring, consistent with TAM signaling’s role in modulating PI3K, MAPK, and STAT3 activity.

Pathway enrichment analysis further supported these findings. The sensitive PDX exhibited dynamic changes, including upregulation of the ERK and AT1R pathways and downregulation of the keratinocyte and EGF pathways, whereas the resistant PDX showed minimal pathway shifts. PI3K/AKT/mTOR signaling emerged as a central node, with significant gene overlaps across enriched pathways. These results indicate that cetuximab sensitivity involves broad transcriptional remodeling, whereas resistance is associated with stable HER signaling and elevated TAM activity. *AXL* expression and PI3K/AKT/mTOR pathway activation may serve as biomarkers for predicting cetuximab response. In line with single-cell and pathway findings, we previously showed the detail of mechanisms of cetuximab resistance in HNSCC (31, 62, 63).

Our findings also provide a rationale for exploring novel EGFR-targeting bispecific antibodies. The Yarden group recently investigated a bispecific antibody targeting both EGFR and AXL in EGFR-mutant non–small cell lung cancer models (64). This dual-targeting approach has the potential to improve current therapeutic strategies by enhancing target specificity, minimizing off-target toxicities, and simultaneously inhibiting complementary resistance pathways. In addition, such approaches may synergistically modulate antitumor immunity and the tumor microenvironment.

It is well established that cetuximab elicits antitumor immune responses through multiple mechanisms, including antibody-dependent cellular cytotoxicity, activation of innate immune responses, and the induction of immunogenic cell death. Because activation of these immune pathways has been associated with improved clinical outcomes, substantial efforts have focused on combining cetuximab with immunotherapeutic approaches (65–68). Although our single-cell analyses did not identify clear immune-related signals associated with response or resistance, our findings may help inform future biomarker-driven therapeutic strategies.

We acknowledge important limitations of this study. The lower-than-anticipated accrual, due in part to the COVID-19 pandemic, limits the statistical power. Thus, we sought samples from collaborators to enable us to analyze correlative objectives without the need for further accrual. Although the study was closed to accrual during the COVID-19 pandemic, we evaluated the ability to meet the primary endpoint to evaluate the relationship between AXL expression and the change in Ki67. After a detailed analysis of the Ki67 and AXL data from the 14 patients who received treatment, as well as the parallel cohort of samples from the University of Michigan’s cetuximab window-of-opportunity TMA, the consensus was that accruing additional subjects needed to reach the target of 36 participants would be highly unlikely to show a significant clinical result.

In conclusion, no correlation between AXL expression at baseline and change in tumor size in response to cetuximab was observed in this small study. We found that the delivery of preoperative cetuximab is safe, and a short course can be utilized to identify patients with HNC responding to single-agent treatment. Further work is needed to understand the multiple mechanisms by which patients with HNC can develop resistance to molecularly targeted drugs such as cetuximab. Window-of-opportunity studies provide an important avenue to assess clinical responses and obtain tissue specimens to help guide mechanistic studies to inform future clinical trial strategies.

## Supplementary Material

Supplemental Figure 1 Change in Ki67 by change in AXL expression.

Supplemental Figure 2 Box and whisker plots for observed difference in Ki67 values for patients with both pre- and post-cetuximab (CTX) AXL expression

Supplemental Table 1 Representativeness of Study Participants

Supplemental Table 2 Correlation of Ki67, AXL expression and tumor size using Kendall's Tau (non-parametric correlation coefficient) for indicated patient populations

## Data Availability

scRNA-seq data files are available via Dryad (https://doi.org/10.5061/dryad.s7h44j1ph). Other data generated in this study are available from the corresponding author upon request.
