# Supplementary material for: Window-of-Opportunity Study Utilizing Preoperative Cetuximab in Patients with Head and Neck Cancer
Source: Cancer Res Commun. 2026 Oct 6;6(10):2325–38. doi: 10.1158/2767-9764.CRC-26-0258 (PMC13639994; doi:10.1158/2767-9764.CRC-26-0258)
Supplement: Supplementary file 1 [file crc-26-0258_supplemental_figure_1_suppsf1.pdf]

Supplemental Figure 1

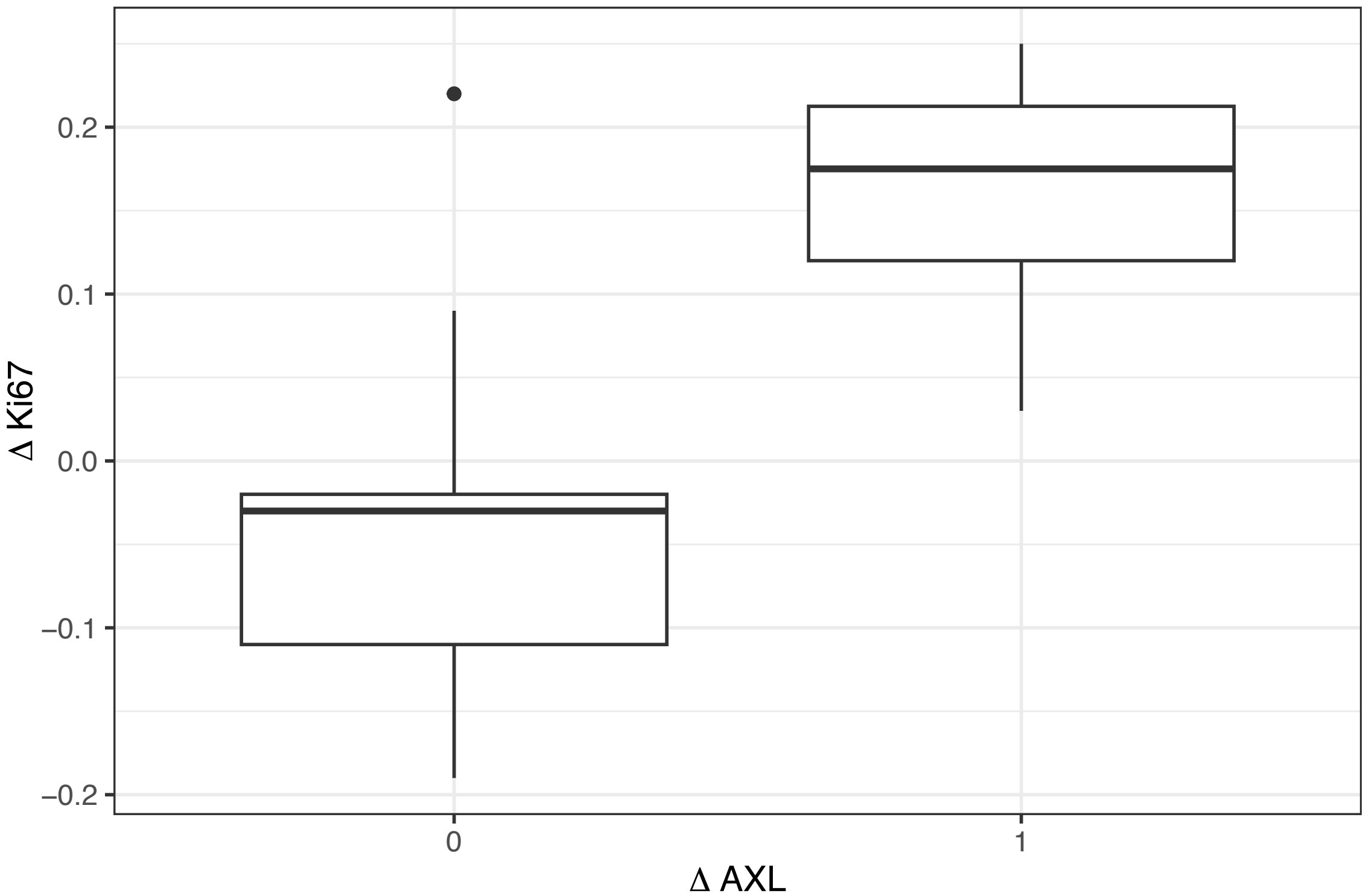

Supplemental Figure 1. Change in Ki67 by change in AXL expression.

Boxplots show the change in Ki67 after cetuximab treatment, grouped by change in AXL immunohistochemistry score from baseline to after cetuximab treatment. Change in AXL was defined as post-treatment AXL score minus baseline AXL score on the 0–4+ scale.
