# Supplementary material for: Window-of-Opportunity Study Utilizing Preoperative Cetuximab in Patients with Head and Neck Cancer
Source: Cancer Res Commun. 2026 Oct 6;6(10):2325–38. doi: 10.1158/2767-9764.CRC-26-0258 (PMC13639994; doi:10.1158/2767-9764.CRC-26-0258)
Supplement: Supplementary file 2 [file crc-26-0258_supplemental_figure_2_suppsf2.pdf]

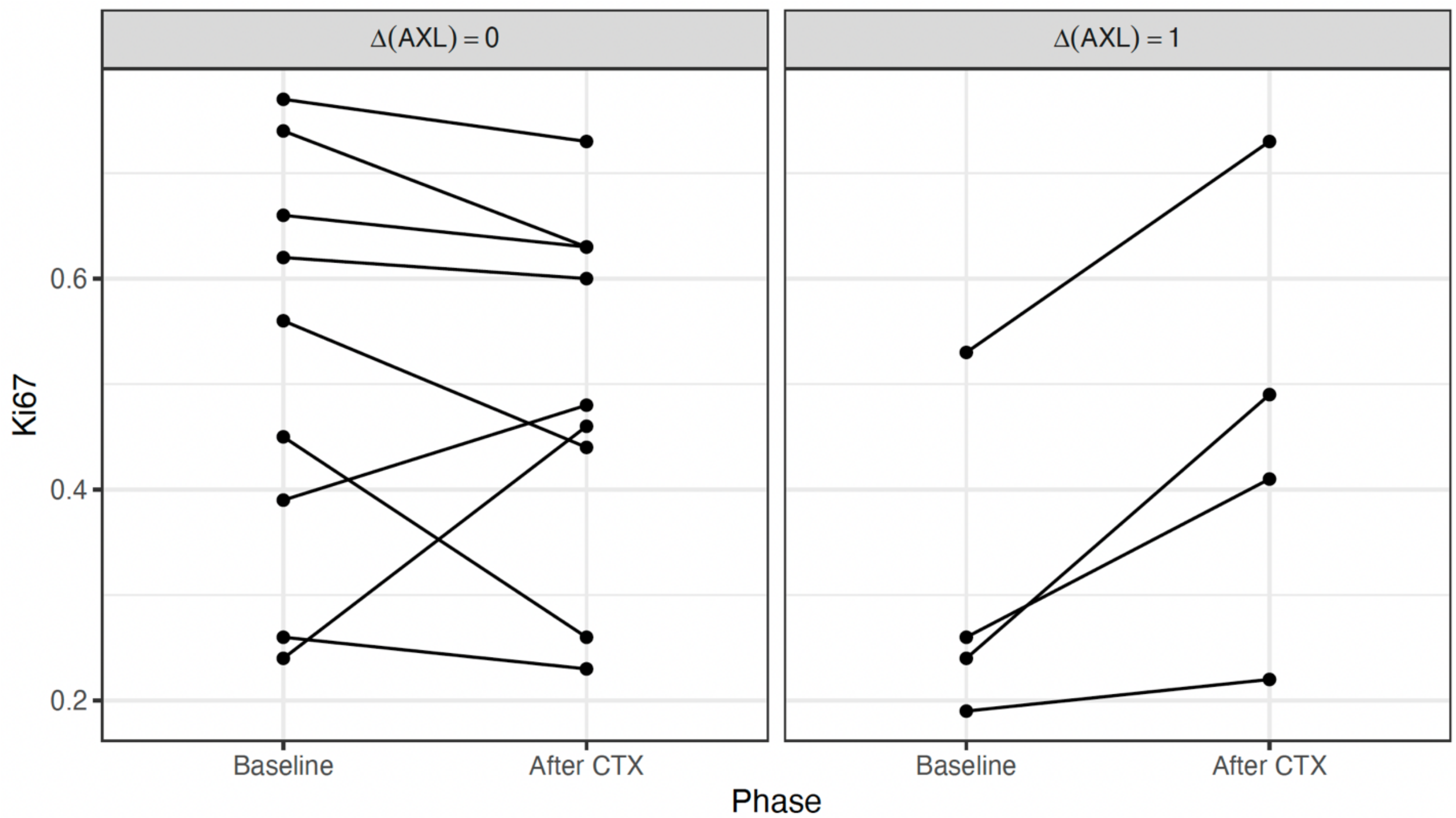

Supplemental Figure 2. Box and whisker plots for observed difference in Ki67 values for patients with both pre- and post-cetuximab (CTX) AXL expression. Patients whose AXL values increased after the introduction of CTX (right frame) had a greater change in percent tumor positive for Ki67 (after CTX (%) minus baseline values) compared to those patients whose AXL values remained the same (right frame) ($p = 0.03$).
